# Digital and AI-assisted approaches across the preeclampsia care pathway: a scoping review

**DOI:** 10.1016/j.xagr.2026.100683

**Published:** 2026-07-31

**Authors:** Ova Emilia, Susaldi Susaldi, Yova Agustini, Meita Dhamayanti, Fanni Hanifa, Hardinsyah Hardinsyah, Triagung Yuliyana, Salma Widya Azhari

**Affiliations:** 1 Department of Obstetrics and Gynecology, Faculty of Medicine, Public Health, and Nursing, Universitas Gadjah Mada, Yogyakarta, Indonesia (Emilia); 2 Faculty of Medicine, Public Health and Nursing, Universitas Gadjah Mada, Yogyakarta, Indonesia (Susaldi, Agustini); 3 Faculty of Health Sciences, Universitas Indonesia Maju, Jakarta, Indonesia (Hanifa, Yuliyana); 4 Faculty of Medicine, Universitas Padjadjaran, Bandung, Indonesia (Dhamayanti, Hanifa); 5 Faculty of Medicine and Nutrition, IPB University, Bogor, Indonesia (Hardinsyah, Yuliyana, Azhari)

**Keywords:** antenatal services, clinical decision support, digital health, health equity, hypertensive disorders of pregnancy, implementation science, maternal health, mobile health, patient education, risk prediction

## Abstract

**OBJECTIVE:**

To map peer-reviewed evidence across four linked functions of digital and AI-assisted preeclampsia care—risk prediction, patient education, remote blood pressure monitoring, and clinician-directed escalation—and determine whether these functions have been evaluated together as an integrated clinical service.

**DATA SOURCES:**

PubMed, Scopus, and ScienceDirect were searched for records published from January 2010 to May 11, 2026.

**STUDY ELIGIBILITY CRITERIA:**

Peer-reviewed original empirical and technical studies of artificial intelligence, machine learning, clinical decision support, telemedicine, mobile health, remote monitoring, digital education, or care escalation for preeclampsia or hypertensive disorders of pregnancy were eligible. Reviews, protocols, conference abstracts without a full report, editorials, and opinion articles were excluded.

**STUDY APPRAISAL AND SYNTHESIS METHODS:**

The review followed PRISMA-ScR and Joanna Briggs Institute guidance. Two reviewers independently screened records. One reviewer charted data and a second independently verified them. Findings were organized by the four predefined functions and synthesized thematically. Formal critical appraisal was not performed; the synthesis therefore describes the evidence map rather than certainty or implementation readiness.

**RESULTS:**

The searches identified 1396 records. After 313 duplicates were removed, 1083 records were screened, and 320 full-text articles were assessed; 286 full-text articles were excluded, and 34 studies conducted in diverse single- and multicountry settings were included. Prediction models reported favorable discrimination estimates, but calibration, independent validation, and equity assessment were inconsistent. Remote monitoring findings were mixed: BUMP 1 did not show earlier clinic-recorded detection of hypertension, whereas some replacement-care models reduced visits or admissions without an observed increase in adverse outcomes. Education studies mainly examined hypothetical responses or communication gaps rather than measured behavior change. Adoption surveys and qualitative implementation studies did not establish clinical effectiveness. No study prospectively evaluated all four functions as a coordinated service; this was treated as a descriptive observation, not evidence of effectiveness or novelty.

**CONCLUSION:**

Component evidence supports staged study, not routine implementation of an integrated pathway. Searching three electronic information sources, excluding gray literature, and not performing formal appraisal may have omitted relevant implementation evidence. Future codesigned prospective studies should evaluate calibration, safety, workload, equity, and maternal and perinatal outcomes.

**AJOG Global Reports at a Glance**

**Why was this study conducted?**

To organize heterogeneous evidence on digital and AI-assisted preeclampsia care across four linked functions: risk prediction, patient education, remote monitoring, and clinician-directed escalation, and to characterize where evidence is direct, limited, or absent.

**Key findings**

Thirty-four peer-reviewed original empirical and technical studies conducted in diverse single- and multicountry settings addressed individual functions, but none prospectively evaluated all four as a coordinated clinical service. Prediction studies often lacked calibration or external validation; monitoring outcomes were mixed; and education and escalation evidence was mostly indirect or early stage.

**What does this add to what is known?**

The four-function framework provides a pragmatic map for staged evaluation while distinguishing component-level evidence from readiness for integrated implementation.

## Introduction

Preeclampsia remains a major cause of maternal and perinatal morbidity and mortality worldwide.^1^^,^^2^ Because it can progress before symptoms become apparent, prevention, and timely recognition depend on identifying risk, communicating actionable information, monitoring blood pressure and symptoms, and ensuring prompt clinical assessment. Evidence-based measures include aspirin prophylaxis for eligible high-risk pregnancies,^1^ angiogenic biomarkers for short-term risk assessment in selected clinical contexts,^3^ and guideline-based surveillance and counseling.^1^^,^^2^ Their effectiveness nevertheless depends on access, continuity, and the capacity of maternity services to act on emerging risk.

Digital care may support some of these functions. Remote blood pressure monitoring can transmit measurements between visits, while mobile applications can provide education, symptom prompts, and contact with maternity services. Individual studies have reported reduced in-person care use or high acceptability in selected settings,^4^, ^5^, ^6^ although the large BUMP 1 trial did not show earlier clinic-recorded detection of hypertension.^7^ Prior reviews likewise describe heterogeneous interventions and outcomes, with limited high-quality evidence and no basis for assuming that benefits transfer across settings.^8^^,^^9^

Artificial intelligence may add risk stratification or decision support, but model performance does not by itself establish clinical utility. Reviews of machine-learning prediction for preeclampsia have identified high reported discrimination alongside substantial heterogeneity, limited external validation, and uncertain transportability.^10^^,^^11^ For example, a retinal-imaging model reported favorable internal performance but still requires independent validation before clinical use.^12^ The unresolved translational question is therefore not whether prediction and digital monitoring exist, but how evidence for distinct functions relates to a safe, accountable care process.

This review uses four linked functions—risk prediction, patient education, remote monitoring, and clinician-directed escalation—as a pragmatic organizing framework. The framework is not proposed as a novel theory or a validated service model. It is used to compare what has been evaluated, what outcomes were measured, and where evidence remains indirect, heterogeneous, or absent. This approach also permits explicit separation of component-level findings from readiness for integration.

### Review aims

This scoping review aimed to map and characterize evidence relevant to digital and AI-assisted preeclampsia care using four distinct questions:

1. Which prediction and risk-stratification approaches have been evaluated, and what is reported about calibration, external validation, and clinical utility?
2. What effects, feasibility findings, and limitations have been reported for remote blood pressure monitoring and digitally supported antenatal care?
3. What evidence addresses patient education, risk communication, adherence, and timely help-seeking?
4. What escalation, governance, implementation, and equity requirements have been examined for clinician-supervised use?

## Methods

### Design and eligibility criteria

This scoping review followed Joanna Briggs Institute methodological guidance and PRISMA-ScR.^13^^,^^14^ No protocol was prospectively registered; eligibility criteria, the charting framework, and synthesis domains were defined before screening. We included peer-reviewed original empirical and technical studies published from January 2010 onward that examined digital or AI-assisted prediction, education, monitoring, decision support, triage, or escalation for preeclampsia or hypertensive disorders of pregnancy. Eligible designs comprised clinical or observational studies, model-development or validation studies, implementation or usability studies, and full technical or conference papers with sufficient methods and results for charting. Reviews, protocols, conference abstracts without a full report, editorials, and opinion articles were excluded.

### Information sources and search strategy

PubMed, Scopus, and ScienceDirect were searched on May 10 to 11, 2026. These sources were selected a priori to provide complementary biomedical indexing, multidisciplinary citation coverage, and publisher full-text coverage within available resources. The scope was deliberately limited to indexed, peer-reviewed original empirical and technical literature; Embase and gray-literature repositories were not searched. The search should therefore be interpreted as a structured map rather than an exhaustive global evidence inventory. Search strings combined terms for preeclampsia or hypertensive disorders of pregnancy; artificial intelligence, machine learning, telemedicine, or digital health; and prenatal or antenatal care. No language or study-design filters were applied at the search stage. Complete platform-specific search strings and execution dates are provided in Supplemental Table 1-4 in accordance with PRISMA-S.^15^

World Health Organization and International Federation of Gynecology and Obstetrics guidance was consulted to contextualize implementation and clinical-care considerations,^16^^,^^17^ but these documents were not treated as eligible primary studies and did not contribute to the included-study count.

### Study selection

**Figure 1:**
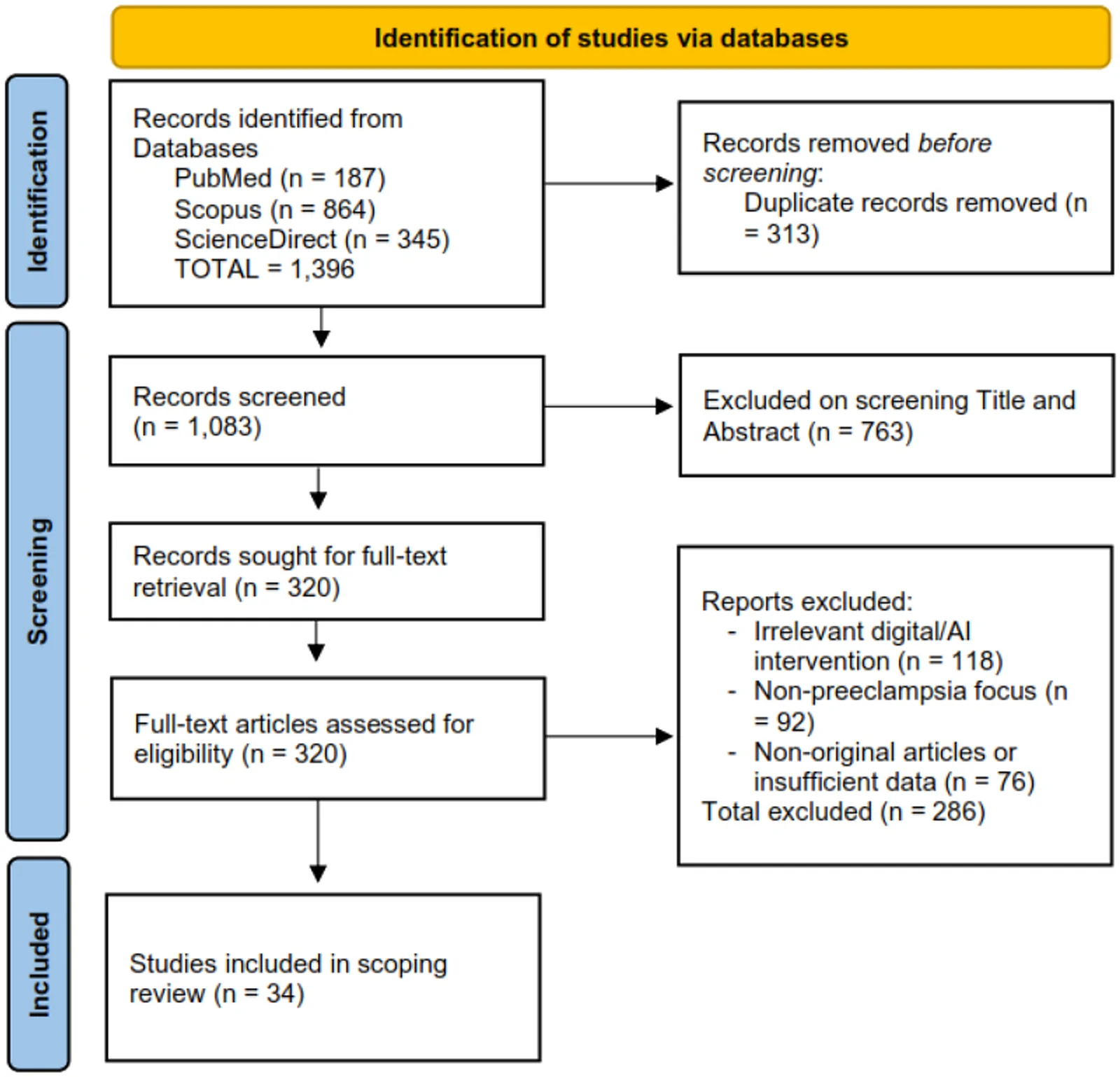
PRISMA flow diagram of study identification, screening, eligibility, and inclusion

Search results were exported to Rayyan for duplicate detection and screening. Two reviewers independently screened titles and abstracts against the eligibility criteria; disagreements were resolved by discussion or consultation with a third reviewer. Two reviewers then assessed the full text of potentially eligible reports. Exclusion reasons were recorded as: no relevant digital or AI component for preeclampsia or hypertensive disorders of pregnancy; no preeclampsia or hypertensive-disorder focus; or nonoriginal source or insufficient data for charting. The selection process is shown in Figure 1.

Emilia. Digital and AI-assisted approaches across the preeclampsia care pathway. AJOG Glob Rep 2026.

### Data charting

A standardized charting form was developed a priori, piloted on five studies, and refined before full application. Extracted items included citation and country; study design and setting; population or data source; technology or intervention; comparator, where applicable; quantitative or qualitative findings; limitations reported by the study; and the pathway function addressed. One reviewer completed charting and a second reviewer independently verified each entry against the source article. Discrepancies were resolved through discussion. A formal inter-rater reliability statistic was not calculated for charting; the independent verification process and this limitation are reported for transparency.

### Synthesis

**Figure 2:**
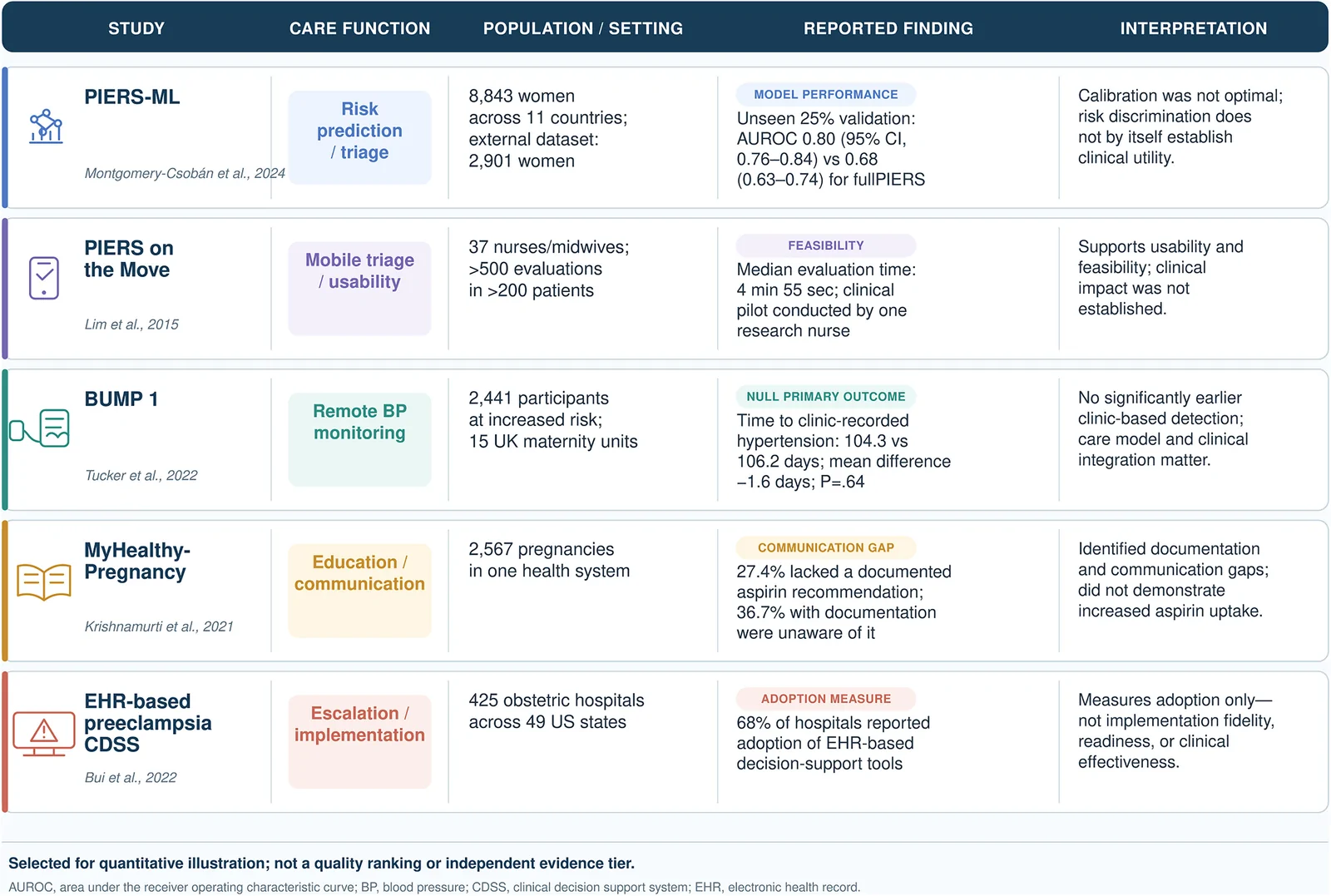
Selected quantitative findings from five included empirical studies Studies were selected because they provided directly extractable findings across different pathway functions; selection does not indicate superior quality or evidentiary status. Values are reported as published, without reanalysis. An initial vector layout was prepared with assistance from ChatGPT (OpenAI; GPT-5-based; accessed July 19, 2026) using author-specified content and published numerical findings. The authors subsequently reviewed and verified all values, labels, interpretations, and visual elements. AUROC, area under the receiver operating characteristic curve; BP, blood pressure; CDSS, clinical decision support system; EHR, electronic health record.

Studies were mapped to the four pathway functions and to cross-cutting implementation and governance considerations using the predefined charting fields. Narrative synthesis compared study design, setting, population, validation approach, outcome type, direction of findings, and reported limitations within and across domains. Contradictory and null findings were retained rather than collapsed into a positive summary. Meta-analysis was not attempted because interventions, populations, designs, and outcomes were heterogeneous. Formal critical appraisal was not performed, consistent with the mapping purpose of a scoping review; consequently, terms implying evidence certainty, maturity, or readiness were avoided. Five empirical studies with directly extractable quantitative findings were selected for descriptive illustration in Figure 2, not as a quality ranking or independent evidence tier.

Emilia. Digital and AI-assisted approaches across the preeclampsia care pathway. AJOG Glob Rep 2026.

### Involvement of patients and maternity service users

Patients and members of the public were not involved in setting the review questions, selecting studies, or interpreting findings. This is a limitation of the review. Patient perspectives reported within included studies were charted where available, and future pathway development should include codesign with pregnant people, families, midwives, and other maternity care professionals.

## Results

### Search results

The searches identified 1396 records: 187 from PubMed, 864 from Scopus, and 345 from ScienceDirect. After removal of 313 duplicates, 1083 titles and abstracts were screened. Of 320 full-text articles assessed for eligibility, 286 were excluded: 118 had no relevant digital or AI component, 92 did not focus on preeclampsia or hypertensive disorders of pregnancy, and 76 were nonoriginal sources or contained insufficient data for charting. Thirty-four studies were included in the evidence map. Study characteristics and pathway mapping are reported in Supplemental Tables 5 and 6.

### Overview of the evidence landscape

The evidence map contained 34 peer-reviewed original empirical and technical studies conducted in diverse single- and multicountry settings. Designs included randomized trials, observational studies, model-development and validation studies, feasibility and technical studies, adoption surveys, and qualitative research. This breadth allowed mapping of multiple care functions but limited direct comparison. No included primary study prospectively evaluated risk prediction, patient education, remote monitoring, and clinician-directed escalation together as a coordinated service in the same population. Given the emerging and heterogeneous nature of the field, this absence was treated as an expected descriptive observation rather than a surprising or independently novel result.

### Risk prediction and decision support

Eighteen studies addressed artificial intelligence or machine-learning prediction, but performance estimates were not directly comparable because populations, prediction windows, outcomes, and validation methods differed. A dynamic electronic-health-record model developed from 58,839 pregnancies across three hospitals within one health system achieved validation AUROCs of 0.808 to 0.834 at its best performing window; positive predictive values remained modest, and transportability beyond that system was not established.^18^ PIERS-ML was developed using data from 8843 women across 11 countries and evaluated in an external sample of 2901 women, with discrimination of approximately 0.80 compared with 0.68 for fullPIERS.^19^ A prospective single-center study of 210 pregnancies reported 96.3% random-forest accuracy, but the small number of outcome events limits precision and generalizability.^20^ In a separate multicountry study, performance of PIERS-ML and fullPIERS declined when reapplied over time after initial assessment, showing that a model’s baseline performance cannot be assumed to persist during serial clinical use.^21^

Emerging approaches also require cautious interpretation. The PROMPT retinal-imaging model analyzed 1812 pregnancies and reported AUROCs of 0.87 for preeclampsia and 0.91 for preterm preeclampsia, but evaluation used cross-validation rather than a separate external test population.^12^ Across studies, calibration was less consistently reported than discrimination, independent prospective validation was uncommon, and subgroup performance by ethnicity, socioeconomic position, or access to the required devices was incompletely examined. These limitations restrict conclusions about clinical utility and equity even when headline discrimination appears favorable.^10^, ^11^, ^12^

For triage and escalation, miniPIERS achieved an AUROC of 0.768 in internal evaluation and 0.713 across external low-resource settings.^22^ In the PIERS on the Move field study, 37 nurses and midwives completed more than 500 evaluations involving more than 200 patients, with a median assessment time of approximately five minutes.^23^ This field study and the related mobile triage application development work support the feasibility of structured risk assessment, not the effectiveness of an end-to-end escalation service.^23^^,^^24^ Similarly, 68% of 425 surveyed US obstetric hospitals reported adoption of electronic decision-support tools for preeclampsia.^25^ This adoption measure indicates diffusion of infrastructure but does not demonstrate risk-stratified use, implementation fidelity, clinical effectiveness, or health-system readiness.

### Remote monitoring, patient education, and implementation

Eighteen studies directly or partially addressed home or remote blood pressure monitoring, representing the largest intervention-focused group in the map, but outcomes were mixed. In BUMP 1, 2441 participants at higher risk of preeclampsia were randomized to self-monitoring with telemonitoring plus usual care or usual care alone. The primary outcome—time to clinic-recorded hypertension—did not differ (104.3 vs 106.2 days; mean difference, −1.6 days; *P*=.64), and maternal and perinatal outcomes were also not significantly different.^7^ By contrast, a recent Australian randomized trial found that replacing some office monitoring with structured remote review reduced outpatient attendances and admissions without an observed increase in adverse maternal or fetal outcomes.^4^ The nonrandomized SAFE@HOME study similarly reported fewer visits, ultrasonography examinations, and hypertension-related admissions, with no detected difference in maternal or perinatal outcomes.^5^ These findings concern different models of care and should not be treated as a single, uniformly positive effect.

A Mississippi feasibility study enrolled 98 Medicaid-eligible participants, of whom 77 used remote monitoring; it assessed feasibility and acceptability rather than comparative effectiveness, and adherence was variable.^6^ Device validation also cannot be assumed: One monitor met international criteria in 51 pregnant participants, although 14% of systolic and 17% of diastolic paired readings differed from reference measurements by more than 10 mm Hg.^26^ Cost analyses of the BUMP trials did not identify differences in health-contact costs.^27^ Collectively, the evidence supports attention to validated devices, training, review schedules, response thresholds, and whether remote contacts replace or add to routine care; it does not establish that telemonitoring alone improves clinical outcomes.

Evidence for preeclampsia-specific digital education and behavior change was thinner and mostly indirect. In an online survey of 1022 pregnant or recently delivered respondents, a hypothetical high-risk result was associated with anticipated, not observed, motivation to follow medication and monitoring recommendations.^28^ In the MyHealthyPregnancy cohort of 2567 pregnancies, 27% of high-risk participants had no documented low-dose aspirin recommendation, and among those with a documented recommendation, 37% were unaware of it.^29^ The app therefore identified communication and documentation gaps; the study did not demonstrate increased aspirin uptake. Prospective studies are needed to determine whether tailored digital communication changes knowledge, adherence, help-seeking, or clinical outcomes.

Implementation evidence highlighted both potential and constraints. A preimplementation P-COACH study based on seven clinician interviews identified possible access benefits alongside liability concerns, alert fatigue, patient anxiety, and the need for workflow-compatible alerts.^30^ Usability testing of the Raabta application with 14 pregnant participants in Pakistan emphasized plain language, voice and visual content, and simpler navigation.^31^ A separate qualitative study of 57 patients, clinicians, decision-makers, and telehealth experts found important differences between public- and private-sector capacity, including internet and device access, staffing, financial resources, language, digital literacy, and responsibility for dashboard review.^32^ These small or context-specific studies deepen understanding of implementation but do not establish effectiveness across settings.

### Cross-function mapping

The four-function map showed evidence relevant to prediction, education, monitoring, and escalation, with substantial overlap because individual studies often addressed more than one domain. However, no included study prospectively evaluated all four functions as a coordinated service with predefined responsibilities and outcomes across the pathway. This observation identifies the absence of end-to-end evaluation; it does not show that integration would improve outcomes or that the components are ready to be combined. Table summarizes the evidence and the limitations that should govern interpretation.

**Table:** Thematic map of evidence across implementation domains

Thematic domain	Mapped studies, *n*^a^	Evidence mapped	Interpretive implication
AI/ML- and CDSS-based risk prediction	20	Model-development and validation studies used electronic health records, biomarkers, retinal imaging, and PIERS-family tools; one survey measured hospital CDSS adoption.	Reported discrimination did not consistently translate to calibration, independent validation, clinical utility, or equitable performance. Adoption is not evidence of effectiveness or implementation preparedness.
Remote/home blood pressure monitoring	18	Randomized trials, observational replacement-care models, feasibility studies, device validation, and cost analyses reported mixed outcomes.	Interpret effects according to the care model, comparator, validated device, clinician-review process, and whether remote contacts replaced or supplemented usual care.
Patient education, adherence, and self-care	15	Evidence included app-based information, risk communication, symptom prompts, and studies of anticipated behavior or communication gaps.	Direct evidence of sustained knowledge, adherence, help-seeking, or clinical benefit was limited; prospective evaluation is required.
Clinician-directed escalation, referral, and triage	20	Evidence included blood-pressure or symptom thresholds, clinician review, PIERS-family risk tools, and mobile decision support.	Feasibility of risk assessment does not establish timely or effective escalation; responsibility, thresholds, response time, and referral capacity must be evaluated.
Implementation, usability, and equity	22	Adoption surveys, small qualitative studies, usability testing, device validation, and cost analyses described workflow and contextual factors.	Transferability depends on workforce, infrastructure, interoperability, language, digital literacy, device access, financing, and local care capacity.

a Domains were not mutually exclusive; one study could contribute to more than one domain. Counts include entries coded as direct, partial, or prototype evidence in Supplemental Table 6; entries coded only as potential relevance were not counted. Counts therefore exceed the 34 included studies when summed.

## Discussion

### Principal findings

Across the 34-study evidence map, evidence was distributed across four linked functions of digital and AI-assisted preeclampsia care, but no included study evaluated all four within one clinician-supervised service. This is a descriptive finding about the organization of an emerging literature, not a claim of conceptual novelty and not evidence that an integrated pathway would be effective. Component-level findings were also uneven: Prediction studies emphasized discrimination more often than calibration or external transportability; monitoring studies reported mixed primary outcomes; education studies largely measured anticipated behavior or communication gaps; and adoption or qualitative implementation studies did not demonstrate clinical benefit.

The synthesis therefore separates evidence for a component from readiness for integration. A model with favorable internal discrimination may still perform poorly in another population or generate low positive predictive values.^10^, ^11^, ^12^^,^^18^ A validated home monitor can provide technically acceptable measurements, but benefit still depends on adherence, review, escalation, and available care.^7^^,^^26^ Likewise, identifying an education gap does not establish that an application changes aspirin use,^28^^,^^29^ and hospital adoption of decision support does not establish appropriate use or improved outcomes.^25^ These distinctions temper the positive findings and define the questions that prospective pathway research must answer.

### Comparison with existing literature

This review is consistent with, rather than dismissive of, prior syntheses. An earlier telemonitoring scoping review found a small, heterogeneous literature dominated by short or nonrandomized studies.^8^ A later systematic review and meta-analysis suggested that clinician-reviewed remote monitoring may reduce antenatal visits or admissions and improve postpartum follow-up but emphasized heterogeneity and the scarcity of high-quality evidence.^9^ Reviews of preeclampsia prediction likewise found that machine-learning models often report favorable discrimination, while external validation, calibration, and transportability remain limited.^10^^,^^11^ The present review contributes an organizational comparison across four care functions; it does not claim to be the first review to identify the broader prediction-to-implementation problem.

The cross-function comparison also clarifies that education and implementation evidence answer different questions from prediction or monitoring trials. Cowan et al^28^ measured anticipated responses to hypothetical risk information, whereas Krishnamurti et al^29^ documented gaps in aspirin recommendation and patient awareness. These findings are consistent with behavior-change and eHealth-literacy frameworks that distinguish access to information from the capability and opportunity to act,^33^^,^^34^ but theoretical consistency is not outcome evidence. Similarly, P-COACH and Raabta studies describe alert burden, liability, workflow, language, and infrastructure constraints,^30^, ^31^, ^32^ but they do not establish safety or effectiveness of deployed services. Evaluation should therefore match claims to the design and outcomes of each evidence strand.

### Clinical and implementation implications

The evidence does not support routine implementation of a unified AI-assisted pathway. Where individual tools are introduced, services should define the clinical purpose, target population, accountable reviewer, review frequency, escalation threshold, backup process, and documentation requirements before deployment. Prediction models require local validation and calibration; blood pressure devices require pregnancy-specific validation; and patient-facing information must provide clear actions without shifting diagnostic responsibility to patients. Alert fatigue and liability cannot be addressed only through interface design: Organizations need staffing, response-time standards, audit processes, and governance for missed or conflicting signals.^16^^,^^30^

Implementation and equity are inseparable. Internet coverage, smartphone and cuff access, data costs, language, digital and health literacy, disability access, interoperability, workforce capacity, and referral availability determine who can use a service and whether an alert can lead to care.^16^^,^^31^^,^^32^ Public- and private-sector differences described in Pakistan illustrate why a tool feasible in one setting may increase burden or exclusion in another.^32^ Digital access should not become an eligibility criterion for high-quality antenatal care, and remote services should include nondigital alternatives and monitoring for differential uptake, false alerts, missed follow-up, and outcomes across population groups.

**Figure 3:**
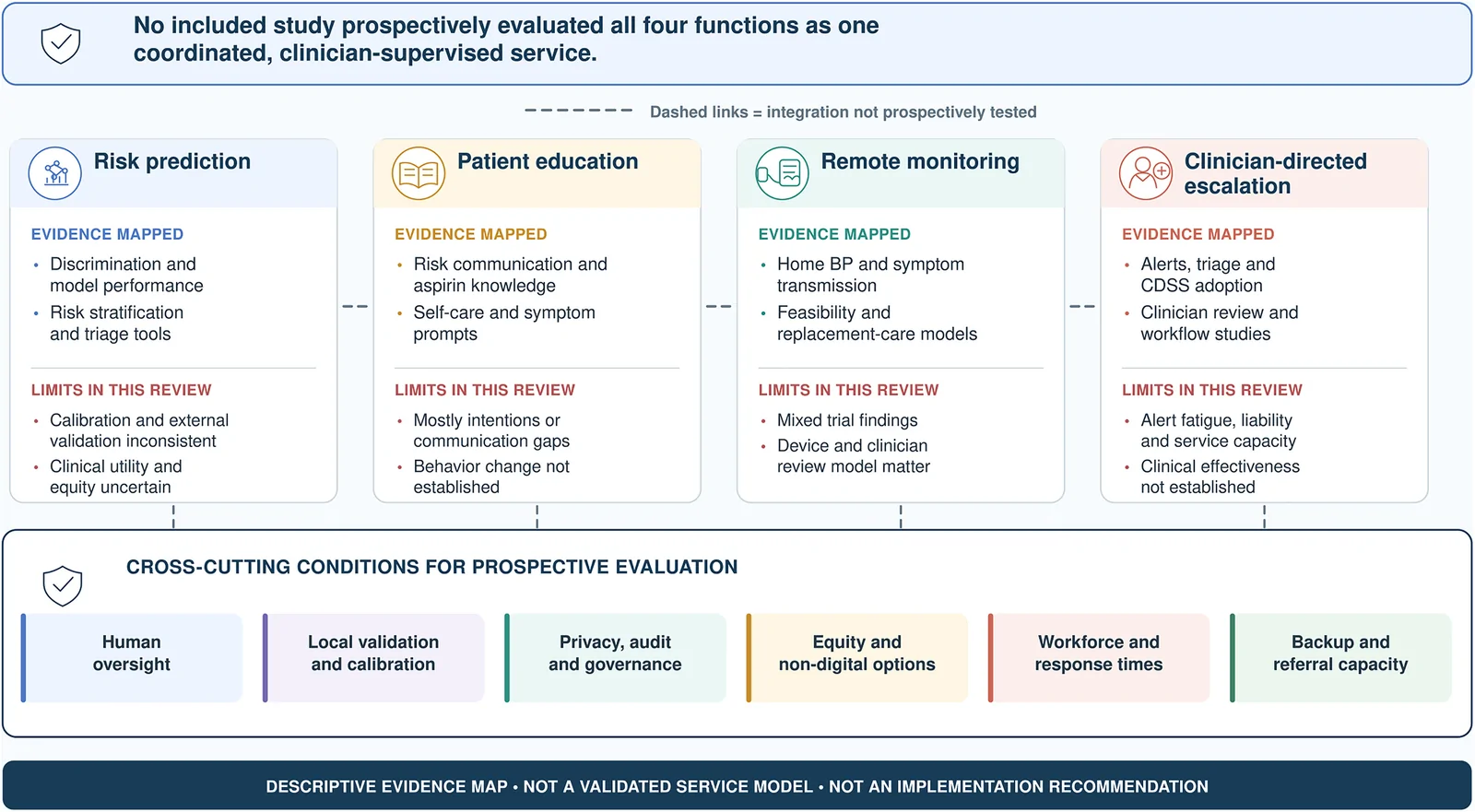
Evidence map of evaluated care functions and untested integration The four panels summarize component-level evidence for risk prediction, patient education, remote monitoring, and clinician-directed escalation. Dashed connectors denote linkages that were not prospectively evaluated together as a coordinated, clinician-supervised service with predefined responsibilities and clinical outcomes. The cross-cutting band summarizes implementation requirements identified in the reviewed evidence and contextual guidance. This descriptive synthesis is not a validated service model or a recommendation for implementation. An initial vector layout was prepared with assistance from ChatGPT (OpenAI; GPT-5-based; accessed July 19, 2026) from the authors’ conceptual framework. The authors subsequently reviewed and verified all concepts, relationships, wording, and visual elements.

Figure 3 visually separates evidence that was evaluated within individual care functions from the linkages that remain untested. Its dashed connections do not imply effectiveness or readiness; they identify where prospective evaluation would be required before prediction, education, monitoring, and escalation could be considered a coordinated service. Cross-cutting requirements shown in the figure were drawn from the implementation evidence and contextual guidance synthesized in this review.

Emilia. Digital and AI-assisted approaches across the preeclampsia care pathway. AJOG Glob Rep 2026.

### Research implications

Research should proceed in stages rather than assuming that integration is the next effective step. Codesign with patients and maternity care professionals should precede feasibility testing of usability, workflow fit, response fidelity, workload, and unintended harms. Prospective comparative studies should then evaluate clinically meaningful outcomes, including time to assessment after an abnormal result, severe maternal morbidity, perinatal outcomes, patient understanding, adherence, anxiety, workload, missed alerts, equity, and costs. Prediction studies should report calibration, decision-curve analysis, external and subgroup validation, and performance over repeated use. Relevant reporting and appraisal frameworks include TRIPOD+AI, PROBAST, DECIDE-AI, and, for trials of AI interventions, CONSORT-AI.^35^, ^36^, ^37^, ^38^

### Strengths and limitations

Strengths include an a priori organizing framework, reproducible platform-specific search strings, independent dual-reviewer screening, second-reviewer verification of charted data, documented exclusion reasons, and inclusion of quantitative, qualitative, feasibility, technical, and implementation designs. Studies conducted in diverse single- and multicountry settings broadened the range of contexts represented, but this does not make the evidence map comprehensive or globally representative.

Several limitations materially affect interpretation. Only PubMed, Scopus, and ScienceDirect were searched; Embase and other potentially relevant bibliographic sources were not included. Gray literature and implementation reports from organizations and programs were not systematically eligible, although WHO and FIGO guidance were used contextually.^16^^,^^17^ These choices may have missed relevant clinical and implementation evidence and may increase selection, publication, and currency bias. Formal critical appraisal was not performed, so the review cannot establish evidence certainty or rank readiness. No formal reliability statistic was calculated for charting, no protocol was registered prospectively, and patients were not involved in the review process. Finally, the Indonesia-based author team’s professional and health-system perspectives may have influenced emphasis despite predefined criteria and independent verification. The findings should therefore be read as a structured, descriptive map rather than a definitive assessment of effectiveness or global implementation readiness.

## Conclusion

Peer-reviewed evidence on digital and AI-assisted preeclampsia care remains fragmented across prediction, education, monitoring, and escalation. Some prediction models and remote-care interventions report favorable component-level findings, but calibration, external validation, mixed trial outcomes, indirect education evidence, and context-specific implementation studies limit claims of readiness. No included primary study evaluated all four functions within one service; this absence does not establish that integration would be beneficial. Because the review searched three electronic information sources and excluded gray literature from the primary map, relevant implementation studies or program reports may have been missed. Future work should use codesign and staged prospective evaluation to determine whether a clinician-supervised pathway can improve maternal and perinatal outcomes without increasing workload, inequity, or safety risk.

## Declaration of AI and AI-assisted technologies in the writing process

During preparation of this revised manuscript, the authors used ChatGPT (OpenAI; GPT-5-based; accessed July 19, 2026) to assist with language refinement, consistency checking, and preparation of an initial vector layout for Figure 2, Figure 3 based on author-defined content. No underlying study data or primary research images were generated or altered. The authors determined the conceptual structure, selected and verified all data and wording, revised the layouts as needed, approved the final figures, and take full responsibility for the content.

## CRediT authorship contribution statement

**Ova Emilia:** Writing – review & editing, Validation, Supervision, Resources, Project administration, Funding acquisition, Conceptualization. **Susaldi Susaldi:** Writing – review & editing, Writing – original draft, Visualization, Project administration, Methodology, Investigation, Formal analysis, Data curation, Conceptualization. **Yova Agustini:** Writing – review & editing, Validation, Methodology. **Meita Dhamayanti:** Writing – review & editing, Validation, Supervision. **Fanni Hanifa:** Writing – review & editing, Validation, Investigation, Data curation. **Hardinsyah Hardinsyah:** Writing – review & editing, Supervision, Resources. **Triagung Yuliyana:** Writing – review & editing, Visualization, Validation, Data curation. **Salma Widya Azhari:** Writing – review & editing, Visualization, Investigation, Data curation.

## Appendix Supplementary materials

Image, application 1

Image, application 2

Image, application 3

## Footnotes

Supplementary material associated with this article can be found in the online version at doi:10.1016/j.xagr.2026.100683.
