# Supplementary material for: Digital and AI-assisted approaches across the preeclampsia care pathway: a scoping review
Source: AJOG Glob Rep. 2026 Jul 31;6(3):100683. doi: 10.1016/j.xagr.2026.100683 (PMC13506530; doi:10.1016/j.xagr.2026.100683)
Supplement: Supplementary file 1 [file mmc1.docx]

**Supplementary File 1. Search Strategy Documentation**

*Digital and AI-Assisted Approaches Across the Preeclampsia Care Pathway: A Scoping Review*

**A. PCC Framework & Concept Map**

The search strategy was structured using the JBI Population-Concept-Context (PCC) framework for scoping reviews. The platform-specific searches operationalized the PCC components below.

| **PCC Component** | **Definition** | **Key Terms Covered** |
| --- | --- | --- |
| Population (P) | Pregnant people receiving antenatal or postpartum care related to preeclampsia or hypertensive disorders of pregnancy | Pregnancy, pregnant women, prenatal care, antenatal care, obstetric care, postpartum hypertension |
| Concept (C) | Digital and AI-assisted approaches across the preeclampsia care pathway | Artificial intelligence, machine learning, clinical decision support, telemedicine, telehealth, mHealth, risk prediction, patient education, remote monitoring, triage, and clinician-directed escalation |
| Context (Cx) | Antenatal, maternity, community, hospital, home, or remote-care settings in any geographic context | Antenatal and maternity care, community obstetric care, single-country and multicountry settings |

**B. Search Log**

| **Database** | **Date Searched** | **Records Retrieved** | **After Deduplication** | **Excluded at T&A screening** | **Full-text articles assessed** | **Included in review** |
| --- | --- | --- | --- | --- | --- | --- |
| PubMed | May 10, 2026 | 187 |  |  |  |  |
| Scopus | May 11, 2026 | 864 |  |  |  |  |
| ScienceDirect | May 11, 2026 | 345 |  |  |  |  |
| TOTAL |  | 1,396 | 1,083 | 763 | 320 | 34 |

*T&A = title and abstract screening.*

**C. Database Search Strings**

**DATABASE 1 — PubMed / MEDLINE (via NLM)**

**Interface:** https://pubmed.ncbi.nlm.nih.gov/ (Advanced Search Builder)

**Date range: January 1, 2010 through May 11, 2026**

**Limits: No language or document-type restrictions were applied at the search stage**

**MeSH note:** Use "Explode" for MeSH terms to capture all narrower terms.

```
(
  "Pre-Eclampsia"[MeSH]
  OR "Hypertension, Pregnancy-Induced"[MeSH]
  OR "HELLP Syndrome"[MeSH]
  OR preeclampsia[tiab]
  OR pre-eclampsia[tiab]
  OR preeclamptic[tiab]
  OR "gestational hypertension"[tiab]
  OR "pregnancy-induced hypertension"[tiab]
)
AND
(
  "Telemedicine"[MeSH]
  OR "Mobile Applications"[MeSH]
  OR "Monitoring, Ambulatory"[MeSH]
  OR "Artificial Intelligence"[MeSH]
  OR "Machine Learning"[MeSH]
  OR "Decision Support Systems, Clinical"[MeSH]
  OR telemedicine[tiab]
  OR telehealth[tiab]
  OR mHealth[tiab]
  OR "mobile health"[tiab]
  OR "digital health"[tiab]
  OR "remote monitoring"[tiab]
  OR "remote patient monitoring"[tiab]
  OR "home blood pressure monitoring"[tiab]
  OR "artificial intelligence"[tiab]
  OR "machine learning"[tiab]
  OR "clinical decision support"[tiab]
  OR "risk prediction"[tiab]
  OR "predictive model*"[tiab]
)
AND
(
  "Prenatal Care"[MeSH]
  OR "Pregnant Women"[MeSH]
  OR "prenatal care"[tiab]
  OR "antenatal care"[tiab]
  OR "obstetric care"[tiab]
)
```

**DATABASE 2 — Scopus (Elsevier)**

**Interface:** https://www.scopus.com (Advanced Search)

**Field tag:** TITLE-ABS-KEY searches title, abstract, and author keywords simultaneously. Use AND/OR/AND NOT. Wildcards: * (zero or more characters).

**Note:** Scopus has no controlled vocabulary. All terms are free-text. Use broader synonyms to compensate.

```
TITLE-ABS-KEY (
  (
    preeclampsia
    OR "pre-eclampsia"
    OR preeclamptic
    OR "gestational hypertension"
    OR "pregnancy-induced hypertension"
  )
  AND
  (
    telemedicine
    OR telehealth
    OR "remote monitoring"
    OR "remote patient monitoring"
    OR "home blood pressure monitoring"
    OR "mobile health"
    OR mHealth
    OR "digital health"
    OR "mobile app*"
    OR "artificial intelligence"
    OR "machine learning"
    OR "clinical decision support"
    OR "risk prediction model*"
  )
  AND
  (
    "antenatal care"
    OR "prenatal care"
    OR pregnan*
    OR "pregnant women"
  )
)
AND PUBYEAR > 2009
```

***Scopus filters***

```
1. Year: 2010–2026 (through May 11, 2026)
2. Subject area: Medicine, Nursing, Health Professions,
   Computer Science, Engineering
Note: No language or document-type restriction was applied at the search stage.
```

**DATABASE 3 — ScienceDirect (Elsevier)**

**Interface:** https://www.sciencedirect.com (Advanced Search)

TITLE(preeclampsia OR "pre-eclampsia" OR "gestational hypertension")

AND

KEY(telemedicine OR telehealth OR mHealth OR "digital health" OR "remote monitoring" OR "artificial intelligence" OR "machine learning")

AND PUBYEAR > 2009

Year range: 2010–2026 (through May 11, 2026)

*Execution note: Each platform-specific string is reproduced as executed; syntax and available fields differed across databases.*

**D. Deduplication Protocol**

After exporting results from all three databases, records were deduplicated using the following steps:

| **No.** | **Action** | **Tool** |
| --- | --- | --- |
| 1 | Export all results to .RIS or .NBIB format from each database | Each database export function |
| 2 | Import all files into the systematic review platform | Rayyan |
| 3 | Run automatic duplicate detection | Rayyan duplicate-detection function |
| 4 | Manually review flagged duplicates — confirm before deleting | Rayyan with manual verification |
| 5 | Record: total before dedup, total after dedup | Search log table (Section B) |
| 6 | Export deduplicated list for title/abstract screening | Rayyan |

**E. Eligibility Criteria for Screening**

| **Criterion** | **Include** | **Exclude** |
| --- | --- | --- |
| Population | Pregnant people, antenatal care users, people at risk of preeclampsia or hypertensive disorders of pregnancy, postpartum patients with pregnancy-related hypertension, and relevant maternity-care professionals or services. | Nonpregnant populations and populations unrelated to pregnancy-related hypertensive care. |
| Disease Focus | Studies addressing preeclampsia, eclampsia, hypertensive disorders of pregnancy, gestational hypertension, or pregnancy-induced hypertension. | General maternal health, antenatal care, or hypertension studies without clear relevance to preeclampsia/HDP. |
| Concept | AI, machine learning, clinical decision support, telemedicine, telehealth, mHealth, digital health, risk prediction, remote or home blood pressure monitoring, patient education, decision support, referral, triage, or clinician-directed escalation related to preeclampsia or hypertensive disorders of pregnancy. | Digital-health or AI studies in pregnancy without relevance to preeclampsia or hypertensive disorders of pregnancy; clinical studies without a digital, predictive, monitoring, educational, decision-support, or pathway component. |
| Context | Antenatal, obstetric, maternity, community, primary-care, hospital, home, or remote-care settings; postpartum settings were eligible when linked to pregnancy-related hypertension. | Nonobstetric settings and postpartum, neonatal, or intrapartum studies unrelated to pregnancy-related hypertension. |
| Study design | Peer-reviewed original empirical and technical studies, including clinical or observational studies, diagnostic or prognostic model studies, intervention studies, implementation or usability studies, and full technical or conference papers with sufficient methods and results for charting. | Reviews, protocols, conference abstracts without a full report, editorials, commentaries, letters, opinion articles, preprints, gray-literature reports, duplicate records, and reports with insufficient data for charting. |
| Language | No language restriction was applied. | No report was excluded solely on the basis of language. |
| Date | Published from January 1, 2010 through May 11, 2026. | Published before January 1, 2010 or after the final search date. |

**F. Notes for PRISMA-ScR Flow Diagram (Figure 1)**

The final PRISMA-ScR flow values used in the manuscript were:

```
IDENTIFICATION
  Records from PubMed:          	187
  Records from Scopus:          	864
  Records from ScienceDirect:   	345
    Total records identified: 	1,396

SCREENING
  Records after deduplication:   	1,083
  Records excluded (T&A screen):   	763
  Records assessed full-text:    	320
Full-text assessed:           	320
Full-text excluded:         	286
    - Irrelevant AI/digital (n=118)
    - No PE/HDP focus (n=92)
    - Non-original/insufficient data (n=76)

INCLUDED
  Studies included in review:       34
```
