# Supplementary material for: Digital and AI-assisted approaches across the preeclampsia care pathway: a scoping review
Source: AJOG Glob Rep. 2026 Jul 31;6(3):100683. doi: 10.1016/j.xagr.2026.100683 (PMC13506530; doi:10.1016/j.xagr.2026.100683)
Supplement: Supplementary file 2 [file mmc2.docx]

**Supplementary Tables S5–S6**

*Digital and AI-Assisted Approaches Across the Preeclampsia Care Pathway: A Scoping Review*

**Supplementary Table S5. Characteristics of the 34 included peer-reviewed original empirical and technical studies**

*The evidence map covers diverse single- and multicountry settings. The final column states each study's descriptive contribution to the evidence map and does not imply comparative quality, certainty, effectiveness, or implementation readiness.*

| **No** | **Citation** | **Country/setting** | **Study design** | **Population/sample** | **Core technology/intervention** | **Contribution to evidence map** |
| --- | --- | --- | --- | --- | --- | --- |
| 1 | Wu et al., 2025 | China; prenatal/early pregnancy care | AI/ML model development and validation using pregnancy cohort data | 1,812 pregnancies before 14 gestational weeks | PROMPT model using retinal photography, retinal vascular parameters, mean arterial pressure, and maternal risk factors | Evaluates early risk prediction using retinal features; relevance is limited to prediction and possible downstream surveillance. |
| 2 | Bui et al., 2022 | United States; hospitals providing obstetric care | Cross-sectional hospital survey analysis | 425 obstetric hospitals across 49 states | EHR-based clinical decision support tools for preeclampsia management | Documents EHR-CDSS adoption; does not measure implementation fidelity, readiness, or clinical effectiveness. |
| 3 | Jhee et al., 2019 | South Korea; tertiary hospital antenatal care | Retrospective EHR-based ML model development | 11,006 pregnant women receiving antenatal care | Prediction model using maternal factors and routine antenatal laboratory data | Evaluates late-onset preeclampsia prediction from routine clinical data; no deployed care pathway was tested. |
| 4 | Li et al., 2021 | China; hospital antenatal care | Retrospective EHR-based ML prediction study | 3,759 pregnancies with antenatal care | EHR-derived clinical parameters from early second trimester; simplified patient-answerable model explored | Evaluates EHR-based early risk prediction; downstream clinical actions were not tested. |
| 5 | Vasilache et al., 2024 | Romania; Cuza Voda Clinical Hospital of Obstetrics and Gynecology, Iasi; first-trimester screening setting | Prospective observational study with machine-learning model development/performance comparison | 210 singleton pregnancies with at least one risk factor for ischemic placental disease; first-trimester screening at 11-13+6 weeks | First-trimester prediction using maternal characteristics, medical/obstetric history, MAP, beta-HCG, PAPP-A, PlGF, and PP-13 | Evaluates first-trimester prediction in a small, single-center high-risk cohort. |
| 6 | Rajkumar et al., 2026 | Australia; three metropolitan teaching hospitals | Non-inferiority, unblinded multicentre randomized controlled trial | Women at high risk of developing preeclampsia; followed to 6 weeks postpartum | Structured remote BP monitoring framework vs office-based surveillance | Evaluates a structured remote-monitoring care model with defined clinical review and escalation. |
| 7 | Campbell et al., 2024 | United Kingdom; antenatal care | Cost analysis of two linked randomized trials | BUMP 1: 2,441 at risk of hypertension; BUMP 2: 850 with hypertension | Self-monitoring BP with mobile telemonitoring system | Reports costs of telemonitoring used in BUMP trials; effectiveness findings derive from the linked trials. |
| 8 | Schor et al., 2024 | United States; prospective multisite nulliparous cohort | ML model development/internal validation using prospective cohort data | 9,124 low-risk nulliparous individuals from nuMoM2b cohort | Risk model at initiation of prenatal care | Evaluates early HDP risk prediction in a prospective cohort with internal validation. |
| 9 | Zhang et al., 2024 | United States; Mississippi; rural/Medicaid/vulnerable population | Prospective pilot cohort feasibility study | 98 enrollees; 77 utilized RPM | Remote patient monitoring of BP until 8 weeks postpartum | Assesses feasibility of remote monitoring in a predominantly Black, rural, Medicaid population. |
| 10 | Tucker et al., 2022 | United Kingdom; 15 hospital maternity units | Unblinded randomized clinical trial | 2,441 pregnant individuals at increased risk of preeclampsia | BP self-monitoring with telemonitoring plus usual care vs usual care | Provides randomized evidence that self-monitoring did not significantly accelerate clinic-recorded hypertension detection. |
| 11 | van den Heuvel et al., 2020 | Netherlands; obstetric care | Prospective case-control digital care-path study | SAFE@HOME n=103; retrospective controls n=133 | Digital platform for daily BP and symptom telemonitoring plus minimal antenatal visit schedule | Evaluates replacement of selected in-person care with structured telemonitoring in a nonrandomized design. |
| 12 | Krishnamurti et al., 2021 | United States; prenatal care app/QI setting | Prospective cohort / quality-improvement initiative using app and EHR cross-reference | 2,567 pregnancies among 2,563 pregnant patients | MyHealthyPregnancy smartphone app with education, monitoring tools, and screening prompts | Identifies documentation and communication gaps around low-dose aspirin recommendations; increased uptake was not demonstrated. |
| 13 | Andersson et al., 2026 | Sweden; antenatal and PE care | Prospective longitudinal feasibility and measurement validation study | 132 normotensive, 40 high-risk, and 87 women with preeclampsia | Anura smartphone app using contactless facial-scan BP monitoring | Assesses acceptability and measurement agreement of contactless smartphone BP monitoring; accuracy was lower in high-risk/PE groups. |
| 14 | Opoku Mensah et al., 2026 | Ghana; low-resource ANC settings | Prospective cohort with points-based clinical prediction/triage tool development | 703 pregnant women attending ANC | Clinical triage score using routine ANC data | Develops a clinical triage score for low-resource antenatal settings; digital implementation was not evaluated. |
| 15 | Khalil et al., 2024 | Multicountry; 21 sites in 6 countries; cfDNA screening cohort | Secondary analysis of prospective multicenter observational cfDNA screening study | 17,520 singleton pregnancies | Artificial neural network and logistic regression using first-visit characteristics plus cfDNA markers | Compares neural-network and logistic-regression prediction using clinical and cfDNA data. |
| 16 | Gopika et al., 2026 | India/USA; engineering prototype/resource-limited settings focus | Prototype development and ML model evaluation using retrospective/synthetic data | Mid-trimester screening; retrospective clinical data plus synthetic patient profiles | Wearable edema detection band + BP sensor + AI risk classifier | Describes a wearable/AI prototype using retrospective and synthetic data; no prospective clinical evaluation. |
| 17 | Munyao et al., 2024 | Kenya; IoT prototype/LMIC focus | IoT device prototype and ML model development | Prototype; future purposive sample planned | Pre-eclampsia watch model with biosensors and cloud processing | Describes an IoT/ML prototype for monitoring and alerts; no real-world clinical evaluation. |
| 18 | Khayatan et al., 2025 | Iran; expert/health informatics setting | Applied study: literature/guideline review + expert questionnaire/Delphi-style consensus | 22 experts including HIM professionals, gynecological surgeons, and midwifery specialists | Information requirement framework for PE CDSS | Defines expert-agreed information requirements for a future CDSS; no clinical model was tested. |
| 19 | Rojas Ramírez et al., 2025 | Peru; Lima; mobile health prototype | Mobile application development and model validation in controlled setting | Pregnant women/end-user testing; small sample not fully extractable from abstract | Mobile app for vital-sign entry, preventive alerts, BI visualizations, and risk comparison | Evaluates a mobile risk-classification prototype in a controlled setting; real-world integration was not examined. |
| 20 | Tran et al., 2023 | Canada; British Columbia Women's Hospital obstetric medicine clinic | Observational quality-improvement/implementation study | 103 people: 44 antepartum and 59 postpartum | Validated home BP monitor with physician interpretation of home readings | Describes implementation of physician-interpreted home BP monitoring in one clinical service. |
| 21 | Canfield et al., 2025 | United States; large academic health center/perinatal care setting | Qualitative pre-implementation study using RE-AIM with hybrid deductive-inductive thematic analysis | Seven perinatal clinicians after 14 invitations; family medicine, obstetrics/gynecology, cardiology, maternal-fetal medicine, and advanced practice nursing perspectives | Adaptation of COACH hypertension application to perinatal care (P-COACH) using home BP monitoring, EHR integration, patient-generated data, visualization, and alerts | Identifies clinician-perceived opportunities and concerns for a proposed perinatal HBPM/CDS adaptation. |
| 22 | Cowan et al., 2024 | United States; online survey | Cross-sectional digital survey | 1,022 pregnant and recently-delivered individuals | Hypothetical early predictive testing and digital survey assessment | Examines hypothetical responses to early predictive testing; observed behavior change was not measured. |
| 23 | Rabby & Doulah, 2025 | Bangladesh/low-resource settings; public dataset | ML model comparison using public patient-record dataset | Publicly available patient records; sample size not clearly stated in abstract | EMR-based ML prediction model | Compares ML models using a public dataset; external and prospective validation were not performed. |
| 24 | Shahil-Feroz et al., 2023 | Pakistan; Karachi; outpatient obstetric setting | Qualitative/formative usability testing using think-aloud and task-completion | 14 pregnant women at high risk of preeclampsia | Raabta app + Bluetooth BP device + clinician dashboard + alerts/self-care messages | Assesses formative usability of a smartphone telemonitoring prototype in a small patient sample. |
| 25 | Montgomery-Csobán et al., 2024 | Multicountry; LMIC and high-income datasets; external validation in UK hospitals | Machine-learning modelling and validation study using pooled prospective datasets | 8,843 women with PE in development/validation; external validation 2,901 women | PIERS-ML maternal risk assessment model | Develops and validates PIERS-ML for short-term maternal risk after preeclampsia diagnosis; clinical utility remains unestablished. |
| 26 | Li et al., 2026 | United States; three NewYork-Presbyterian hospitals | Retrospective multisite EHR cohort ML model development and external validation | 58,839 pregnancies | Dynamic prediction using longitudinal EHR data | Develops dynamic EHR-based short-term prediction; prospective workflow integration was not evaluated. |
| 27 | Arkerson et al., 2023 | United States; two academic institutions; postpartum setting | Multisite randomized controlled trial | Postpartum patients with pregnancy-related hypertension/HDP | Remote BP monitoring through web-enabled smartphone platform vs in-office BP check | Evaluates postpartum remote BP monitoring with protocol-based follow-up; antenatal applicability is limited. |
| 28 | Shahil-Feroz et al., 2024 | Pakistan; public and private sector hospitals | Qualitative descriptive implementation study using CFIR | 57 semi-structured interviews with patients, obstetricians, decision-makers, and telehealth experts | Raabta smartphone-based telemonitoring program | Describes stakeholder-perceived implementation barriers and facilitators across public and private sectors. |
| 29 | Yang et al., 2025 | Multicountry prospective datasets; Americas, sub-Saharan Africa, South Asia, Europe, Oceania | Multicountry prospective observational model performance study | 8,843 women diagnosed with preeclampsia | Serial/consecutive use of PIERS-ML and fullPIERS models | Evaluates serial use of PIERS-ML/fullPIERS and shows performance decline after baseline assessment. |
| 30 | Lim et al., 2015 | South Africa; Tygerberg Hospital, Cape Town and Frere Maternity Hospital, East London; LMIC-focused mHealth setting | Iterative usability testing and pilot clinical feasibility evaluation | 37 nurses and midwives evaluated the tool; pilot use by one research nurse included more than 500 evaluations on more than 200 patients | PIERS on the Move mHealth platform combining miniPIERS risk assessment with Phone Oximeter and standardized antenatal assessment | Assesses usability and pilot feasibility of mHealth triage; clinical outcome effectiveness was not established. |
| 31 | Aquino et al., 2022 | Canada; high-risk obstetrical clinic | Qualitative descriptive study | 7 patients at high risk for PE and 5 healthcare providers | Telemonitoring needs assessment for BP self-care and clinical management | Identifies patient and clinician requirements for telemonitoring in a small qualitative study. |
| 32 | Oben et al., 2023 | United States; pregnancy BP device validation | Device validation study using AAMI/ESH/ISO protocol | 51 pregnant participants across normotensive, hypertensive without proteinuria, and preeclampsia subgroups | Ideal Life BP Manager that automatically transmits BP to medical record independent of internet access | Validates a remote BP device in pregnancy; care-process and clinical outcomes were not evaluated. |
| 33 | Payne et al., 2014 | Multicountry LMICs: Brazil, Fiji, Pakistan, South Africa, Uganda; external fullPIERS dataset | Prospective multicountry cohort model development and external validation | 2,081 women with HDP in LMICs; external validation 1,300 women | miniPIERS risk prediction model using simple clinical variables | Develops and externally validates miniPIERS for adverse maternal risk after HDP presentation. |
| 34 | Dunsmuir et al., 2014 | LMIC/community-based care; PIERS on the Move application development | mHealth application development/technical implementation paper | Community-based healthcare provider users; application designed for pregnant women with PE | mHealth apps collecting symptoms/clinical measures including smartphone-connected pulse oximeter; REDCap synchronization | Describes technical development of an mHealth triage application; clinical effectiveness was not evaluated. |

**Supplementary Table S6. Descriptive mapping of included studies across the preeclampsia digital-care continuum**

*✓ = directly evaluated; Partial = function present but not primary; Potential = conceptual relevance only and not evaluated; Prototype = developmental or technical work without clinical-effectiveness evidence; blank = not addressed. Domains may overlap. Coding supports evidence mapping only and is not a quality, effectiveness, maturity, or readiness rating.*

| **No** | **Citation** | **AI/ML/CDSS component** | **Digital component** | **Risk prediction** | **Patient education** | **Remote monitoring** | **Clinician-directed escalation** | **Implementation/usability** | **Evidence-map interpretation** |
| --- | --- | --- | --- | --- | --- | --- | --- | --- | --- |
| 1 | Wu et al., 2025 | Deep-learning retinal feature extraction + machine-learning prediction | PROMPT model using retinal photography, retinal vascular parameters, mean arterial pressure, and maternal risk factors | ✓ |  |  | Potential |  | Evaluates early risk prediction using retinal features; relevance is limited to prediction and possible downstream surveillance. |
| 2 | Bui et al., 2022 | EHR-based CDSS adoption; not AI predictive modeling | EHR-based clinical decision support tools for preeclampsia management |  |  |  | ✓ | ✓ | Documents EHR-CDSS adoption; does not measure implementation fidelity, readiness, or clinical effectiveness. |
| 3 | Jhee et al., 2019 | Decision tree, naïve Bayes, SVM, random forest, stochastic gradient boosting, logistic regression | Prediction model using maternal factors and routine antenatal laboratory data | ✓ |  |  | Potential |  | Evaluates late-onset preeclampsia prediction from routine clinical data; no deployed care pathway was tested. |
| 4 | Li et al., 2021 | LR, RF, SVM, XGBoost | EHR-derived clinical parameters from early second trimester; simplified patient-answerable model explored | ✓ |  |  | Potential |  | Evaluates EHR-based early risk prediction; downstream clinical actions were not tested. |
| 5 | Vasilache et al., 2024 | Decision tree, naive Bayes, support vector machine, and random forest algorithms | First-trimester prediction using maternal characteristics, medical/obstetric history, MAP, beta-HCG, PAPP-A, PlGF, and PP-13 | ✓ |  |  | Potential |  | Evaluates first-trimester prediction in a small, single-center high-risk cohort. |
| 6 | Rajkumar et al., 2026 | Rules/protocol-based telemonitoring; no AI model | Structured remote BP monitoring framework vs office-based surveillance |  | ✓ | ✓ | ✓ | ✓ | Evaluates a structured remote-monitoring care model with defined clinical review and escalation. |
| 7 | Campbell et al., 2024 | Automated telemonitoring alerts; no AI model | Self-monitoring BP with mobile telemonitoring system |  | Partial | ✓ | ✓ | ✓ | Reports costs of telemonitoring used in BUMP trials; effectiveness findings derive from the linked trials. |
| 8 | Schor et al., 2024 | Random forest with recursive feature elimination | Risk model at initiation of prenatal care | ✓ |  |  | Potential |  | Evaluates early HDP risk prediction in a prospective cohort with internal validation. |
| 9 | Zhang et al., 2024 | Telemonitoring workflow; no AI model | Remote patient monitoring of BP until 8 weeks postpartum |  | Partial | ✓ | ✓ | ✓ | Assesses feasibility of remote monitoring in a predominantly Black, rural, Medicaid population. |
| 10 | Tucker et al., 2022 | Automated app messaging; no AI model | BP self-monitoring with telemonitoring plus usual care vs usual care |  | Partial | ✓ | ✓ | ✓ | Provides randomized evidence that self-monitoring did not significantly accelerate clinic-recorded hypertension detection. |
| 11 | van den Heuvel et al., 2020 | Protocol/rules-based dashboard alerts; no AI model | Digital platform for daily BP and symptom telemonitoring plus minimal antenatal visit schedule |  | Partial | ✓ | ✓ | ✓ | Evaluates replacement of selected in-person care with structured telemonitoring in a nonrandomized design. |
| 12 | Krishnamurti et al., 2021 | Rules-based app screening; no AI model | MyHealthyPregnancy smartphone app with education, monitoring tools, and screening prompts | Partial | ✓ | Partial | Potential | ✓ | Identifies documentation and communication gaps around low-dose aspirin recommendations; increased uptake was not demonstrated. |
| 13 | Andersson et al., 2026 | Smartphone-based contactless BP estimation; no PE risk model | Anura smartphone app using contactless facial-scan BP monitoring |  | Partial | ✓ | Potential | ✓ | Assesses acceptability and measurement agreement of contactless smartphone BP monitoring; accuracy was lower in high-risk/PE groups. |
| 14 | Opoku Mensah et al., 2026 | Logistic regression-derived score; not digital AI | Clinical triage score using routine ANC data | ✓ |  |  | ✓ | Potential | Develops a clinical triage score for low-resource antenatal settings; digital implementation was not evaluated. |
| 15 | Khalil et al., 2024 | Feedforward neural network; logistic regression comparator | Artificial neural network and logistic regression using first-visit characteristics plus cfDNA markers | ✓ |  |  | Potential |  | Compares neural-network and logistic-regression prediction using clinical and cfDNA data. |
| 16 | Gopika et al., 2026 | Logistic regression, decision tree, gradient boosting; CDSS risk output | Wearable edema detection band + BP sensor + AI risk classifier | ✓ |  | Potential | Potential | Prototype | Describes a wearable/AI prototype using retrospective and synthetic data; no prospective clinical evaluation. |
| 17 | Munyao et al., 2024 | Naïve Bayes model selected after comparison with DT, KNN, LR, SVM, ANN | Pre-eclampsia watch model with biosensors and cloud processing | ✓ |  | ✓ | ✓ | Prototype | Describes an IoT/ML prototype for monitoring and alerts; no real-world clinical evaluation. |
| 18 | Khayatan et al., 2025 | CDSS data requirements; no deployed model | Information requirement framework for PE CDSS | ✓ |  |  | Potential | ✓ | Defines expert-agreed information requirements for a future CDSS; no clinical model was tested. |
| 19 | Rojas Ramírez et al., 2025 | Random Forest risk-level model | Mobile app for vital-sign entry, preventive alerts, BI visualizations, and risk comparison | ✓ | ✓ | Partial | ✓ | Prototype | Evaluates a mobile risk-classification prototype in a controlled setting; real-world integration was not examined. |
| 20 | Tran et al., 2023 | Clinician-managed HBPM; no AI model | Validated home BP monitor with physician interpretation of home readings |  | Partial | ✓ | ✓ | ✓ | Describes implementation of physician-interpreted home BP monitoring in one clinical service. |
| 21 | Canfield et al., 2025 | EHR-integrated clinical decision support/data visualization; no AI predictive model | Adaptation of COACH hypertension application to perinatal care (P-COACH) using home BP monitoring, EHR integration, patient-generated data, visualization, and alerts |  | ✓ | ✓ | ✓ | ✓ | Identifies clinician-perceived opportunities and concerns for a proposed perinatal HBPM/CDS adaptation. |
| 22 | Cowan et al., 2024 | No model; examines behavioral impact of predictive testing | Hypothetical early predictive testing and digital survey assessment | ✓ | ✓ | Potential | Potential | ✓ | Examines hypothetical responses to early predictive testing; observed behavior change was not measured. |
| 23 | Rabby & Doulah, 2025 | LR, Passive Aggressive Classifier, Extra Trees, SVM, CatBoost, Random Forest | EMR-based ML prediction model | ✓ |  |  | Potential |  | Compares ML models using a public dataset; external and prospective validation were not performed. |
| 24 | Shahil-Feroz et al., 2023 | Rules-based NICE-informed alert algorithm; no AI model | Raabta app + Bluetooth BP device + clinician dashboard + alerts/self-care messages |  | ✓ | ✓ | ✓ | ✓ | Assesses formative usability of a smartphone telemonitoring prototype in a small patient sample. |
| 25 | Montgomery-Csobán et al., 2024 | Random forest-based ML model; compared with fullPIERS logistic model | PIERS-ML maternal risk assessment model | ✓ |  |  | ✓ |  | Develops and validates PIERS-ML for short-term maternal risk after preeclampsia diagnosis; clinical utility remains unestablished. |
| 26 | Li et al., 2026 | Extreme gradient boosting models for 1-, 2-, and 4-week windows | Dynamic prediction using longitudinal EHR data | ✓ |  |  | Potential |  | Develops dynamic EHR-based short-term prediction; prospective workflow integration was not evaluated. |
| 27 | Arkerson et al., 2023 | Protocol/rules-based triage; no AI model | Remote BP monitoring through web-enabled smartphone platform vs in-office BP check |  | ✓ | ✓ | ✓ | ✓ | Evaluates postpartum remote BP monitoring with protocol-based follow-up; antenatal applicability is limited. |
| 28 | Shahil-Feroz et al., 2024 | Rules-based telemonitoring workflow; no AI model | Raabta smartphone-based telemonitoring program |  | ✓ | ✓ | ✓ | ✓ | Describes stakeholder-perceived implementation barriers and facilitators across public and private sectors. |
| 29 | Yang et al., 2025 | PIERS-ML and fullPIERS model evaluation | Serial/consecutive use of PIERS-ML and fullPIERS models | ✓ |  |  | ✓ |  | Evaluates serial use of PIERS-ML/fullPIERS and shows performance decline after baseline assessment. |
| 30 | Lim et al., 2015 | miniPIERS risk model plus decision-support recommendations; Phone Oximeter integration; no AI/ML model | PIERS on the Move mHealth platform combining miniPIERS risk assessment with Phone Oximeter and standardized antenatal assessment | ✓ |  | Partial | ✓ | ✓ | Assesses usability and pilot feasibility of mHealth triage; clinical outcome effectiveness was not established. |
| 31 | Aquino et al., 2022 | Desired CDSS/action prompts; no deployed AI | Telemonitoring needs assessment for BP self-care and clinical management |  | ✓ | ✓ | ✓ | ✓ | Identifies patient and clinician requirements for telemonitoring in a small qualitative study. |
| 32 | Oben et al., 2023 | Remote BP device validation; no AI model | Ideal Life BP Manager that automatically transmits BP to medical record independent of internet access |  |  | ✓ | Potential | ✓ | Validates a remote BP device in pregnancy; care-process and clinical outcomes were not evaluated. |
| 33 | Payne et al., 2014 | Logistic regression clinical prediction model; not AI but used within later mHealth triage tools | miniPIERS risk prediction model using simple clinical variables | ✓ |  |  | ✓ | Potential | Develops and externally validates miniPIERS for adverse maternal risk after HDP presentation. |
| 34 | Dunsmuir et al., 2014 | miniPIERS risk score + decision tree treatment/referral recommendations | mHealth apps collecting symptoms/clinical measures including smartphone-connected pulse oximeter; REDCap synchronization | ✓ |  | Partial | ✓ | ✓ | Describes technical development of an mHealth triage application; clinical effectiveness was not evaluated. |
